# Supplementary material for: WordCommentsAnalyzer: A windows software tool for qualitative research
Source: F1000Res. 2018 Sep 4;7:536. Originally published 2018 May 3. [Version 2] doi: 10.12688/f1000research.14819.2 (PMC6137411; doi:10.12688/f1000research.14819.2)
Supplement: Supplementary file 3 [file f1000research-7-17614-s0002.tgz › b2a79496-4ada-47c4-9682-a99ce867836d.zip!/Eight Iranian Health Journals/Journal of Knowledge and Health_21.docx]

© Journal of Knowledge and Health. This work is licensed under CC BY 4.0 (https://creativecommons.org/licenses/by/4.0/).

Volume & Issue: دوره 8، شماره 1: 1392

Title: رابطه سلامت روانی، رضايت از زندگی و کمال‌گرايی با سردرد ميگرن

http://knh.shmu.ac.ir/index.php/site/article/view/18

مقدمه: سردرد ميگرن يکی از بيماری‌های شايع است. هدف از مطالعه حاضر، بررسی ارتباط سلامت روانی، كمال‌گرايي و رضايت از زندگی با سردرد ميگرن در دانشجويان دانشگاه پيام نور اهواز است. مواد و روش‌ها: در اين مطالعه توصيفی، 286 نفر (225 دختر و 61 پسر) به‌صورت تصادفی طبقه‌اي انتخاب شدند. برای جمع‌آوري داده‌ها از پرسش‌نامه‌های سلامت روانی، رضايت از زندگی، کمال‌گرايی و سردرد ميگرن استفاده شد. تجزيه‌و‌تحليل داده‌ها با استفاده از رگرسيون چندگانه انجام شد. نتايج: نتايج مطالعه نشان داد که متغيرهای سلامت روانی و رضايت از زندگی با سردرد ميگرن همبستگی منفی معنادار و کمال‌گرايی با سردرد ميگرن دانشجويان مورد مطالعه، همبستگی مثبت معناداری دارد. ضريب همبستگی چندگانه بين متغيرهای پيش‌بين و سردرد ميگرن برابر 417/0 است. همچنين نتايج مطالعه نشان داد که سلامت روانی و رضايت از زندگی، پيش‌بين‌کننده سردرد ميگرن می‌باشند.نتيجه‌گيری: سردرد ميگرن ازطریق سلامت روانی و رضايت از زندگی قابل پیش‌بینی است.

Title: بررسی بی‌خطری و كارايي انفوزیون دوز پایین پروپوفول در مقایسه با میدازولام، طی اعمال کولونوسکوپی و کلانژیوپانکراتوگرافی رتروگراد

http://knh.shmu.ac.ir/index.php/site/article/view/20

مقدمه: از پروپوفول برای ایجاد سدیشن در بیماران كانديد كولونوسكوپي و كلانژيوپانكراتوگرافي استفاده شده است، ولی دوز مشخصی از دارو که كارايي لازم را همراه با ایمنی کامل جهت تجویز در خارج از اتاق عمل داشته باشد، در دست نیست. در این پژوهش، بی‌خطری و كارايي پروپوفول با دوز شروع mg/kg 8/0 و دوز ادامه µg/kg/min25 با میدازولام وریدی mg/kg1/0 مقایسه شد. مواد و روش‌ها: در اين کارآزمایی بالینی، تعداد 140 بیمار طی 7 ماه به‌طور تصادفی در دو گروه پروپوفول و میدازولام بررسی شدند. متغیرهای فشارخون سیستولیک، تعداد ضربان قلب و تعداد تنفس در دقيقه و اشباع هموگلوبین از اکسیژن (SPO2) جهت مقایسه بی‌خطر‌بودن پروپوفول و پاسخ‌های سوماتیک و اتونوم بیمار به تحریک جراحی و همچنین طول ریکاوری، سرعت ترخیص از بیمارستان، یادآوری وقایع حین عمل و رضایتمندی پزشك معالج، جهت مقایسه كارايي استفاده شد. نتایج: گروه پروپوفول از ثبات بیشتر فشارخون سیستولیک برخوردار بود (05/0P<). تغییرات تنفسی دو گروه یکسان بود. هیپوکسی و آپنه در هیچ بیماری دیده نشد. میانگین زمان ریکاوری و زمان لازم برای ترخیص بیمار در گروه پروپوفول، به‌صورت مشخص پایین‌تر بود. میانگین سایر متغیرها تفاوت معنادار نداشت و در هر دو گروه یکسان بود. نتیجه‌گیری: پروپوفول با دوز فوق می‌تواند جایگزینی مناسب برای میدازولام در کولونوسکوپی و کلانژیوپانکراتوگرافی رتروگراد باشد و مزیت آن نسبت‌به میدازولام، ثبات بیشتر فشار‌خون شریانی، کاهش زمان ریکاوری و ترخیص سریع‌تر بیمار از بیمارستان است.

Title: تأثير مصرف مکمل اسید آمینۀ شاخه‌دار (BCAA) بر پروتئین واکنشگر-C (HS-CRP) پس از یک جلسه فعالیت مقاومتی در فوتبالیست‌ها

http://knh.shmu.ac.ir/index.php/site/article/view/38

مقدمه: مطالعه حاضر به‌منظور بررسی اثر مصرف مکمل اسیدهای آمینه شاخه‌دار (Branched- Chain Amino Acid) بر پروتئین واکنشگر-C سرم فوتبالیست‌ها متعاقب یک جلسه فعالیت مقاومتی صورت گرفت. مواد و روش‌ها: 20 فوتبالیست جوان در قالب طرح دو سوکور، در یک جلسه فعالیت مقاومتی شامل 7حرکت ورزشی با 3ست 8-10 تکراری شرکت کردند. آزمودنی‌ها به 2 گروه (10 نفری) مکمل و کنترل (شبه‌دارو) به‌صورت تصادفی تخصیص داده شدند. 30 دقیقه قبل از اجرای آزمون ورزشی به آزمودنی‌ها، اسیدهای آمینه شاخه‌دار (200 میلی‌گرم به‌ازای هر کیلو‌گرم وزن بدن) یا دکسترین (شبه‌دارو) داده شد. نمونه‌های خونی 30 دقیقه قبل، بلافاصله، 1، 2 و 24 ساعت پس از فعالیت مقاومتی سنگین از ورید آنتی‌کوبیتال آزمودنی‌ها جمع‌آوری شد. برای تجزیه‌و‌تحلیل یافته‌های تحقیق از تحلیل واریانس با اندازه‌گیری‌های مکرر و آزمون تعقیبی بونفرونی استفاده گردید. نتایج: در اندازه‌گیری مقادیر پایه HS-CRP، 30 دقیقه قبل از فعالیت ورزشی، تفاوتی بین گروه مکمل و شبه‌دارو وجود نداشت (05/0P>). افزایش معناداری بین مقادیر پایه HS-CRP قبل و بلافاصله، 1،2 و 24 ساعت پس از فعالیت مقاومتی وجود داشت (05/0P<). درحالی‌که مصرف مکمل BCAA باعث کاهش معنادار HS-CRP 24 ساعت پس از فعالیت مقاومتی در مقایسه با گروه شبه‌دارو شده بود (05/0P<). نتیجه‌گیری: مکمل BCAA باعث کاهش معنادار شاخص التهابی HS-CRP به‌دنبال فعالیت مقاومتی می‌شود. بنابراین می‌توان گفت که مصرف مکمل BCAA، بیومارکر التهاب را کاهش دهد که با فعالیت مقاومتی مرتبط است.

Title: نقش پیش‌بین شادکامی، خوش‌بینی و وضعیت جمعیت‌شناختی در اقدام به رفتارهای مرتبط با سلامت

http://knh.shmu.ac.ir/index.php/site/article/view/39

مقدمه: باتوجه به اهمیت سبک زندگی در ارتقای سلامت، هدف این پژوهش بررسی نقش پیش‌بین شادکامی، خوش‌بینی و وضعیت جمعیت‌شناختی، در انجام رفتارهای مرتبط با سلامت در بین دانش‌آموزان دبیرستانی شهرستان سمنان بود. مواد و روش‌ها: در یک طرح توصیفی مقطعی، 217 نفر (104 پسر و 113 دختر) از دانش‌آموزان دبیرستانی شهر سمنان به شيوه نمونه‌گيری خوشه‌ای دومرحله‌ای انتخاب شدند و پرسش‌نامه اطلاعات جمعیت‌شناختی، پرسش‌نامه شادکامی آکسفورد (OHI)، آزمون بازنگری‌شده جهت‌گیری زندگی (LOT-R) و مقیاس ارتقای سلامت نوجوانی (AHP) را تکمیل کردند. تحلیل داده‌ها با استفاده از همبستگی و رگرسیون چندگانه به‌روش گام‌به‌گام با نرم‌افزار SPSS انجام گرفت. نتایج: یافته‌ها نشان داد که بین شادکامی، خوش‌بینی، وضعیت اجتماعی- اقتصادی، با اقدام به رفتارهای مرتبط با سلامت، همبستگی مثبت معناداري وجود دارد (001/0P<). شادکامی، خوش‌بینی، وضعیت اجتماعی- اقتصادی مي‌توانند به‌طور معناداری اقدام به رفتارهای مرتبط با سلامت دانش‌آموزان دبیرستانی را پیش‌بینی کنند (001/0P<) و درمجموع 54% اقدام به رفتارهای مرتبط با سلامت را تبیین مي‌نمایند (54/0R2=). در این مدل، سن و جنس در تبیین اقدام به رفتارهای مرتبط با سلامت، نقش معناداری نداشتند (05/0<P). نتیجه‌گیری: شادکامی، خوش‌بينی و وضعیت اجتماعی- اقتصادی، در اقدام به رفتارهای مرتبط با سلامت دانش‌آموزان دبیرستانی مهم هستند. پیامد اصلی این مطالعه، تدوین مداخلاتی به‌منظور بهبود این عوامل به‌عنوان مبنایی مهم در اقدام به رفتارهای مرتبط با سلامت و ارتقای سلامت دانش‌آموزان دبیرستانی است.

Title: ارتقاي درك از بيماري مولتيپل اسكلروزيس (ام‌اس) و حيطه‌هاي آن در بيماران شركت‌كننده در گروه حمايتي همتايان

http://knh.shmu.ac.ir/index.php/site/article/view/40

مقدمه: درک از بيماری، عقايد بيمار از بيماري مي‌باشد که در تصميم‌گيری بيمار برای انجام رفتارهای مؤثر در ارتباط‌با بيماری و دستيابی به نتايج مثبتی چون تطابق با بيماری و بهبود عملکرد تأثير دارد. از‌آنجايی‌که تابه‌حال در ايران برروی درک از بيماری در بيماران ام‌اس پژوهشی انجام نشده است، پژوهش حاضر تأثير گروه همتايان را بر درک از بيماری ام‌اس و حيطه‌های آن در مبتلايان به ام‌اس بررسی مي‌کند. مواد و روش‌ها: اين پژوهش یک پژوهش نیمه‌تجربي قبل و بعد بود که برروي 33 بيمار مبتلا به ام‌اس در سه گروه حمايتي همتا (10 نفر درگروه مردان، 11 نفر در گروه زنان و 12 نفر در گروه مختلط زن و مرد) به‌مدت 8 هفته و هر هفته 2 ساعت انجام شد. سطح درک از بیماری ام‌اس با استفاده از "پرسش‌نامه تعديل‌شده درک از بيماری" که 75 عبارت دارد و ضريب آلفای کرونباخ آن 80/0 بود، قبل و بعد از تشکيل گروه‌ها سنجيده شد. نتايج: ميانگين نمرات درک از بيماری ام‌اس در بيماران شرکت‌کننده در گروه همتايان بعد از مداخله گرچه افزایش یافته بود، ولی اين افزايش به‌لحاظ آماری معنادار نبود. در بین سه گروه، شرکت در گروه همتايان سبب ارتقاي سطح درک از بيماری در گروه مختلط (01/0=P) شده بود. نتيجه‌گيری: نتايج پژوهش نشان داد كه شرکت درگروه همتايان سبب افزايش درک از بيماری در مبتلايان به ام‌اس مي‌شود و باتوجه به اين امر که حمايت اجتماعي، احساس تعلق در افراد ايجاد مي‌کند و تطابق بهتر با حوادث زندگي را به دنبال دارد، لذا ضرورت دارد مداخلاتي برپايه حمايت اجتماعي و ارتقاي سطح درك از بيماري براي مبتلايان به ام‌اس طراحي شود. همچنین نتايج پژوهش را مي‌توان در حيطه‌های آموزش گروه‌های علوم پزشکی و مديريت بالينی به‌کارگرفت.

Title: ارزیابی ظرفيت آنتي‌اكسيدان و ضدّ‌التهابی عصاره الكلي بابونه، توت، ختمی، گل گاوزبان و رزماری

http://knh.shmu.ac.ir/index.php/site/article/view/41

مقدمه: یافتن ترکیبات جدید با منشأ گیاهی در درمان، تخفیف و پیشگیری از بیماری‌ها، در سال‌هاي اخير مورد توجه محققان است. هدف مطالعه حاضر بررسي توان آنتی‌اکسیدانی و اثرات ضدّالتهابی گیاه‌های گل‌گاوزبان، گل ختمي، بابونه، برگ درخت توت و رزماري مي‌باشد که به کرار در طب سنتی استفاده مي‌شوند. مواد و روش‌ها: در این بررسی میزان توان آنتی‌اکسیدانی گیاهان با ارزيابي احيا يون مس (Cupric Assay)، قدرت ضدّ‌التهابی آنها با آزمون ممانعت از دنچوره‌شدن پروتئين سرم، صورت پذیرفت. نتايج: عصاره برگ درخت توت، بابونه و گل گاوزبان دارای بیشترین اثرات آنتی‌اکسیدانی، عصاره رزماری، بابونه و گل ختمی داراي بیشترین اثرات ضدّالتهابی می‌باشند و دو گیاه بابونه و رزماری دارای اثرات توأم آنتی‌اکسیدانی و ضدّ‌التهابی هستند. نتیجه‌گیری: استفاده از روش‌های غربالگری همانند روش‌های فوق‌الذکر فرصتی برای یافتن ترکیبات فعال دارای چند اثر متفاوت را فراهم خواهد کرد.

Title: روان‌سنجی مقیاس گلاسكوي اصلاح‌شده پالما وكوك در بيماران بستري در بخش مراقبت‌هاي ويژه توسط ارزیابان آموزش‌ندیده

http://knh.shmu.ac.ir/index.php/site/article/view/43

مقدمه: آرام‌سازی کمتر یا بیشتر از حد نیاز، باعث ايجاد مشکلات بسياري برای بیماران بخش مراقبت‌های ویژه می‌شود. ابزارهای روا و پایایی برای مانیتورینگ سطح آرام‌سازی این بیماران وجود ندارد. هدف از این مطالعه، تعیین اعتبار و پايايي مقياس گلاسكوي اصلاح‌شده پالما وكوك در بيماران بزرگسال بستري در بخش مراقبت‌هاي ويژه توسط ارزیابان آموزش‌ندیده می‌باشد. مواد و روش‌ها: پس از ترجمه نسخه انگلیسی مقیاس گلاسکو اصلاح‌شده پالما و کوک به زبان فارسی، با استفاده از روش ارزیابی كيفيت زندگی بین‌المللي، هشت نفر ارزیاب (شامل: محقق اصلی، شش نفر پرستار و پزشک مقیم بیهوشی)، 70 بیمار را به‌طور مستقل با استفاده از مقیاس گلاسکو اصلاح‌شده پالما و کوک مورد ارزيابي قرار دادند. محقق اصلی علاوه‌براین دو نمره، امتیاز بیمار براساس مقیاس آنالوگ چشمی و مقیاس ریچموند جهت ارزیابی اعتبار مقیاس مذکور را ثبت می‌نمود. ارزیابان، درمورد مقیاس گلاسکو اصلاح‌شده پالما و کوک آموزش قبلی دریافت نکرده ‌بودند. نتایج: ضریب همبستگی درون‌گروهي (ICC) برای مقیاس گلاسكو اصلاح‌شده پالما و كوك در گروه آموزش‌ندیده 898/0 بود و ضریب کاپا که نشان‌دهنده توافق بین ارزیابان می‌باشد 894/0 بود. همچنین ارتباط مثبت و معناداری (001/0P<) بین امتیاز مقیاس گلاسكو اصلاح‌شده پالما و كوك و مقیاس آنالوگ چشمی (91/0r=) و مقیاس آرام‌سازی آژیتاسیون ریچموند (897/0 r=) و مقیاس گلاسکو کوما (920/0r=) وجود داشت. نتیجه‌گيری: اعتبار و پایایی مقیاس گلاسكو اصلاح‌شده پالما وكوك برای ارزیابی سطح آرام‌سازی در بیماران بزرگسال بستری در بخش مراقبت‌های ویژه، حتی توسط ارزیابان آموزش‌ندیده، تأیید می‌گردد.

Title: ارزیابی جامعه به‌منظور شناسایی و تعیین حجم مشکلات مرتبط با سلامت

http://knh.shmu.ac.ir/index.php/site/article/view/45

مقدمه: نقش ارزیابی جامعه عبارت از شناسایی عوامل تأثیرگذار درسلامت جمعیت و تعیین میزان دسترسی به منابع درون آن جامعه می‌باشد. اين مطالعه با هدف شناسايي مشكلات دو منطقه مختلف از شهرستان شاهرود و انتخاب مشكلي براي بررسي عميق‌تر و تعیین حجم مشکل در دو منطقه آن انجام شده است. مواد و روش‌ها: در این مطالعه با کمک مدل 8 مرحله‌ای ارزیابی جامعه‌ی کارولینای شمالی مشکلات منطقه مهدی‌آباد و شهر بسطام شناسایی و اولویت‌بندی شد. سپس از بین مشکلات، مشکل سلامت روان به‌عنوان یک مشکل اولویت‌دار مشترک در دو منطقه انتخاب و به‌منظور تعیین حجم این مشکل تعداد 450 نفر در منطقه‌ی مهدی‌آباد و 407 نفر از منطقه‌ی بسطام به‌روش خوشه‌ای سیستماتیک تصادفی انتخاب و با استفاده از پرسشنامه‌ی دموگرافیک و پرسشنامه‌ی‌ سلامت عمومی (GHQ-28) و پرسشنامه 5 سؤالي وضعيت تندرستي WHO مورد سنجش قرار گرفتند. یافته‌ها: مشکل سلامت روان ششمین مشکل اولویت‌دار در مهدی‌آباد و چهارمین مشکل اولویت‌دار در بسطام بود. شیوع کلی سلامت روان نامطلوب (حدود اطمینان) در منطقه‌ی مهدی‌آباد و بسطام به‌ترتیب 9/43% (5/48-3/39) و 1/25% (3/29-9/20) بود. درصد مردان و زنان با وضعيت روان و تندرستي غيرطبيعي در منطقه مهدي‌آباد بيشتر از منطقه بسطام بود. از نظر خرده مقياس‌هاي علايم جسماني و علايم اضطرابي منطقه‌ي بسطام و از نظر خرده مقياس‌هاي كاركرد اجتماعي و افسردگي منطقه مهدي‌آباد وضعيت نامناسب‌تري داشتند. نتیجه‌گیری: فرآیند ارزیابی جامعه یک ابزار کاملاً مفید جهت نیازسنجی، شناسایی و حل مشکلات جامعه بوده و به‌کارگیری آن جهت ارتقاء وضعیت سلامت و رفاه هر جامعه‌ای اکیداً توصیه می‌شود.
